# Want a State Prosecuting Agent

**Published:** 1897-04

**Authors:** 


THE physicians, druggists and dentists of Maine want a State prosecuting agent to see to the enforcement of the laws applying to medicine, pharmacy and dentistry. The bill that aims at the establishing of such an office was drawn up by Dr. F. Hanson. The Boston *Herald* says:

"The first section of this bill provides for the appointment by the Governor of an officer who shall be the prosecuting agent for the State in cases of infraction of medical, dental and pharmaceutical laws. He shall serve for a term of four years. It shall be the agent's duty to investigate all violations of the laws pertaining to these professions, and he shall prosecute all offenders against these laws. He shall have the power to call upon the several county attorneys and the attorney-general for aid in prosecuting offenders, and shall himself be possessed of all the powers delegated to the sheriffs of the various counties. He may serve writs and make arrests, and shall also have the power of a coroner. For his services he shall receive no direct compensation, but shall be paid in fines collected."

If this passes in Maine, and works well, it would be a good idea for other States to imitate.

